# Insulin signaling in PCOS/PMOS: molecular insights and clinical implications

**DOI:** 10.3389/fendo.2026.1955884

**Published:** 2026-09-25

**Authors:** Abhishek Goel, Ashna Grover, Skand Shekhar

**Affiliations:** 1 Clinical Research Branch (CRB), National Institute of Environmental Health Sciences (NIEHS), National Institutes of Health (NIH), Research Triangle Park, NC, United States; 2 National Institute of Diabetes and Digestive and Kidney Diseases (NIDDK), National Institutes of Health (NIH), Bethesda, MD, United States; 3 Section on Neuroendocrinology and Reproduction, Brigham and Women's Hospital, Harvard Medical School, Boston, MA, United States

**Keywords:** follicles, hyperandrogenism, hyperinsulinism, insulin resistance, metabolic syndrome, ovaries, polycystic ovary syndrome

## Abstract

Insulin signaling in polycystic ovary syndrome (PCOS) or Polyendocrine Metabolic Ovarian Syndrome (PMOS) involves selective pathway alterations that link metabolic disease to reproductive pathology. In metabolic pathways, impaired PI3K/Akt signaling limits insulin-dependent GLUT4-mediated glucose intake, while ovarian steroidogenic responses to insulin are preserved. Compensatory pancreatic β-cell insulin secretion and reduced hepatic insulin clearance lead to hyperinsulinemia, suppressing sex hormone-binding globulin, increasing free androgen bioavailability, and amplifying ovarian androgen production. In ovarian theca cells, hyperinsulinemia results in excess androgen secretion, while granulosa cells demonstrate reduced glucose uptake, estradiol production, and follicular maturation largely due to metabolic insulin resistance. Collectively, these alterations across hepatic, skeletal muscle, adipose, and lipid-metabolic tissues reinforce an insulin-dependent hyperandrogenemia further amplified by obesity and hyperleptinemia. Resultantly, women with PCOS/PMOS frequently present with both reproductive dysfunction and metabolic syndrome. Therefore, treatment approaches are tailored to individual patient circumstances and generally include lifestyle modification to assist with weight management and improve insulin sensitivity, which may be bolstered by the use of insulin sensitizers like metformin and incretin analogs (GLP1As) that promote weight loss, attenuate insulin resistance, and suppress hepatic gluconeogenesis. Other insulin-sensitizing agents hold promise as evidence evolves. In this review, we summarize how altered insulin signaling plays a prominent role in PCOS/PMOS pathophysiology and discuss the current evidence for lifestyle and pharmacologic strategies to improve the metabolic and reproductive dysfunction in this syndrome.

## 1 Introduction

Polycystic ovary syndrome (PCOS), recently renamed to polyendocrine metabolic ovary syndrome (PMOS) (1), is a common endocrine disorder affecting an estimated 8–13% of reproductive-aged women worldwide, yet up to 70% of cases remain undiagnosed (2). The 2023 International PCOS guidelines diagnose adults with PCOS/PMOS when at least two features are present after excluding other causes: oligo-anovulation, hyperandrogenism, and polycystic ovarian morphology or elevated anti-Müllerian hormone (AMH). In adolescents, diagnosis requires both ovulatory dysfunction and hyperandrogenism, as ultrasound morphology and AMH have limited specificity during adolescence (3). Selective alterations in systemic insulin signaling play a key role in the metabolic and reproductive dysfunction pathognomonic of PCOS/PMOS.

Despite their high burden, metabolic dysfunction, obesity, and insulin resistance are not formal diagnostic criteria, reflecting disease complexity and evolving understanding (3, 4). Insulin resistance can present in PCOS/PMOS independent of BMI, as insulin clamp-based studies demonstrate reduced insulin sensitivity even after adjusting for obesity (5–7). Importantly, such insulin resistance is selective, with metabolic insulin signaling impaired in systemic tissues, whereas the hyperinsulinemia-induced hyperandrogenic and steroidogenic actions are unaffected.

Hyperinsulinemia in PCOS/PMOS is a combined effect of compensatory pancreatic β-cell secretion and impaired insulin clearance (8). After secretion into the portal circulation, insulin acts on hepatic and target tissues and is substantially cleared by the liver. In PCOS/PMOS, reduced metabolic clearance rate of insulin (MCRI) prolongs systemic insulin exposure independent of insulin secretion, creating positive feedback that worsens both reproductive and metabolic outcomes (9). Moreover, hyperinsulinemia suppresses hepatic sex hormone-binding globulin (SHBG), increases free androgen availability, and directly augments ovarian steroidogenic signaling, linking metabolic dysfunction to hyperandrogenism, follicular arrest, and anovulation (10, 11). Systemic insulin resistance and hyperinsulinemia result in increased risk of metabolic comorbidities, including dysglycemia, metabolic syndrome, metabolic dysfunction-associated fatty liver disease (MAFLD), and obstructive sleep apnea (12–14). Thus, understanding how insulin signaling is altered at both the cellular and systemic levels is essential for explaining the reproductive, endocrine, and cardiometabolic burden of the syndrome.

We first review selective insulin signaling as the cellular basis for metabolic resistance with preserved ovarian steroidogenic sensitivity. We then examine systemic insulin handling across target tissues, and discuss clinical manifestations and phenotypes linked to PCOS/PMOS-associated insulin resistance and opportunities for targeted therapies.

This review examines how selective insulin resistance and compensatory hyperinsulinemia connect ovarian, hepatic, skeletal muscle, adipose, and neuroendocrine physiology, supporting the view of PCOS/PMOS as a polyendocrine syndrome rather than purely a reproductive disorder (1).

Targeted PubMed searches combining PCOS/PMOS terms with hyperinsulinism, insulin resistance, insulin sensitivity, metabolic syndrome, and menstrual disturbance terms. Management-focused searches were supplemented by PubMed, Embase, and Web of Science strategies addressing obesity, exercise, metformin, dyslipidemia, and obstructive sleep apnea in PCOS/PMOS.

## 2 Selective insulin resistance in PCOS

Insulin is a peptide hormone with a wide range of actions across organ systems, including pleiotropic effects on glucose and lipid handling, vascular function, growth, immune function, and reproductive effects (15–17). The key metabolic disturbance in PMOS is selective insulin resistance with hyperinsulinemia rather than global disruption in insulin signaling. Insulin-stimulated metabolic signaling is impaired in somatic tissues and in some reproductive tissues, whereas ovarian steroidogenic responses are preserved, thereby amplifying androgen excess. This tissue-specific pattern explains how hyperinsulinemia coexists with metabolic insulin resistance while promoting ovarian androgen excess (18–21).

At the pathway level, insulin resistance in PMOS/PCOS appears to arise largely from post-binding signaling defects (22). Insulin receptor activation engages PI3K/Akt to support AS160-dependent GLUT4 translocation and glucose uptake, while parallel MAPK/ERK-related signaling supports growth and differentiation (23, 24). In PMOS/PCOS, inhibitory IRS-1 Ser312 phosphorylation can blunt PI3K/Akt signaling, reducing insulin-stimulated glucose uptake despite high upstream insulin exposure (25). Reduced GLUT4 expression in endometrial epithelium from hyperinsulinemia in women with PMOS/PCOS further supports tissue-specific metabolic impairment in reproductive tissues.

However, ovarian insulin resistance is inconsistent across cell types. Granulosa cells exhibit impaired metabolic signaling, whereas theca cells retain insulin-sensitive steroidogenic output (**Figure 1A**). In theca cells, preserved insulin responsiveness and reduced MEK/ERK restraint favor CYP17A1-linked androgen biosynthesis (20, 21). In granulosa cells, impaired PI3K/Akt signaling limits glucose uptake and cellular energy availability, contributing to failed follicular maturation. Thus, selective insulin resistance underlies how hyperinsulinemia can worsen both metabolic and reproductive dysfunction without requiring impairment of the same insulin pathway in every tissue (26, 27).

**Figure 1:**
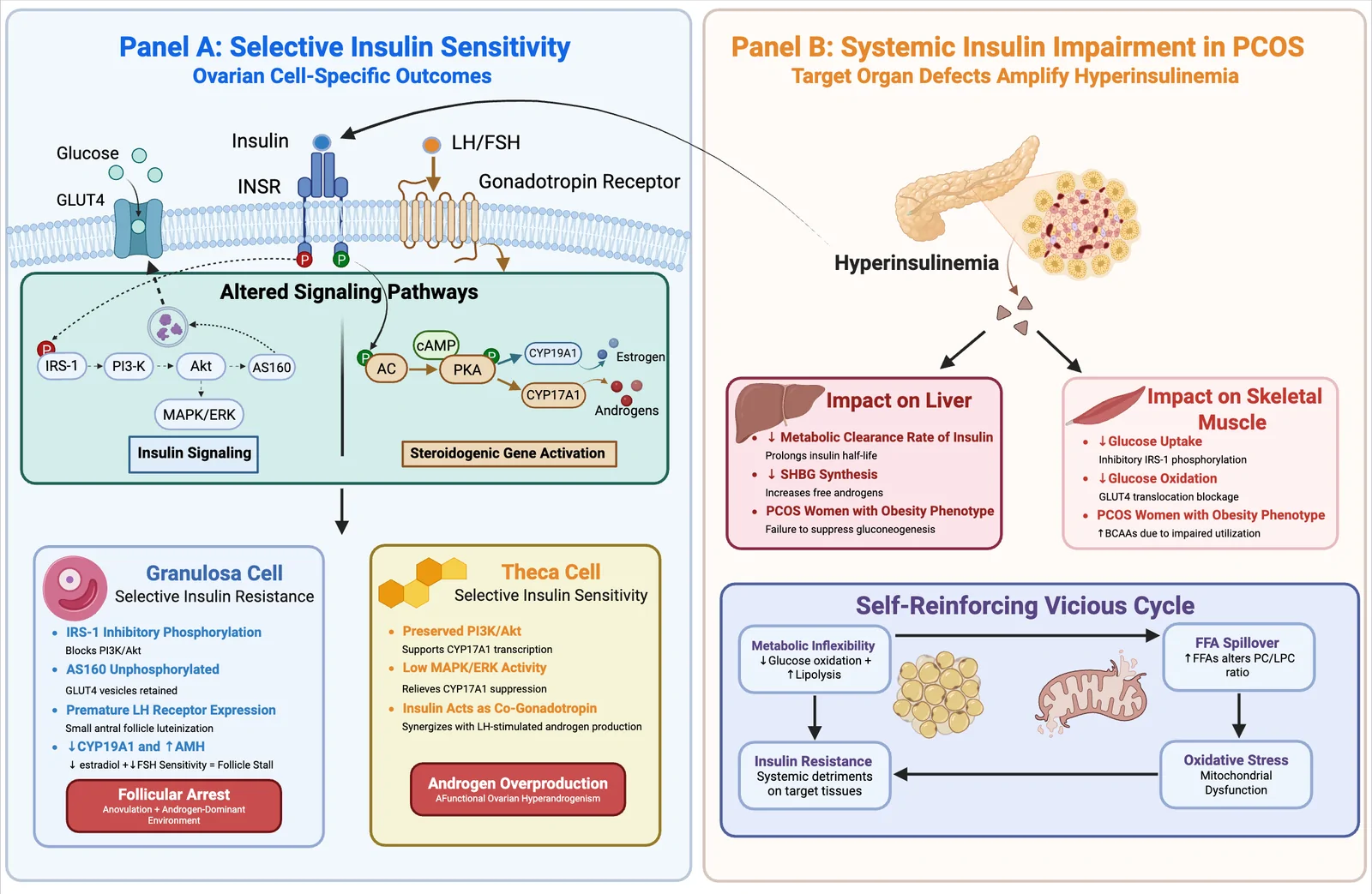
Insulin signaling and systemic insulin feedback in PCOS. **(A)** Cellular basis of selective insulin resistance in ovarian tissue. Insulin and LH share intracellular signaling pathways, with pleiotropic impacts in theca and granulosa cells: theca cells retain steroidogenic insulin sensitivity (hyperandrogenemia), whereas granulosa cells show impaired metabolic insulin signaling (ovulatory dysfunction). **(B)** Systemic effects of differential insulin signaling. Pancreatic beta-cell insulin hypersecretion and reduced hepatic insulin clearance result in hyperinsulinemia. In parallel, liver, skeletal muscle, and lipid pathways exacerbate selective insulin resistance and androgen bioavailability. AC, adenylyl cyclase; AMH, anti-Mullerian hormone; AS160, Akt substrate of 160 kDa; BCAA, branched-chain amino acid; cAMP, cyclic adenosine monophosphate; CYP17A1, cytochrome P450 family 17 subfamily A member 1; CYP19A1, aromatase; FFA, free fatty acid; FSH, follicle-stimulating hormone; GLUT4, glucose transporter type 4; INSR, insulin receptor; IR, insulin resistance; IRS-1, insulin receptor substrate 1; LH, luteinizing hormone; MAPK/ERK, mitogen-activated protein kinase/extracellular signal-regulated kinase; MCRI, metabolic clearance rate of insulin; PC/LPC, phosphatidylcholine/lysophosphatidylcholine; PI3K/Akt, phosphoinositide 3-kinase/protein kinase B; PKA, protein kinase A; SHBG, sex hormone-binding globulin.

## 3 Altered insulin signaling in PCOS and its impacts on target tissues

In PMOS/PCOS, hyperinsulinemia reflects both increased beta-cell demand and impaired insulin clearance. Systemic insulin resistance increases pancreatic β-cell demand, leading to compensatory hypersecretion. At the same time, reduced hepatic insulin extraction lowers MCRI and prolongs insulin exposure (9), resulting in fasting euglycemia accompanied by impaired postprandial glucose tolerance and metabolic dysfunction.

The liver is both a metabolic target and regulator of systemic insulin exposure. Reduced hepatic insulin clearance increases the duration and impact of insulin signaling across tissues. Hyperinsulinemia also suppresses hepatic sex hormone-binding globulin (SHBG) secretion, increasing the bioavailable fraction of circulating androgens (28). Diazoxide has been shown to increase circulating SHBG after suppression of insulin secretion, supporting the notion that hyperinsulinemia contributes directly to the low-SHBG state in PCOS/PMOS (29). Increased free androgen availability then feeds back into the PCOS/PMOS phenotype by worsening hyperandrogenic symptoms and disordered follicular development.

Skeletal muscle represents another major site of impaired insulin-mediated glucose disposal. Euglycemic-hyperinsulinemic clamp studies have shown reduced insulin-mediated glucose processing in women with PCOS/PMOS compared with weight-matched controls (5, 30, 31). The mitogenic action of insulin may be attenuated in PCOS/PMOS independent of adiposity. In one report, investigators noted a severe reduction in extracellular-signal-regulated kinase (ERK) 1/2 pathway stimulation through increased phosphorylation leading to reduced mitogenesis (32). Current evidence suggests that the defect is primarily upstream of mitochondrial metabolism: insulin signaling abnormalities impair glucose uptake and oxidation, whereas mitochondrial mass and oxidative phosphorylation can remain relatively preserved in primary myotubes (25, 33, 34). Skeletal muscle dysfunction is potentially modifiable through interventions that improve proximal insulin signaling and glucose disposal.

Insulin appears to promote androgen secretion by the adrenals through insulin receptors on the surface of adrenocortical cells to upregulate multiple steps in the steroidogenesis cascade (35), independently of ACTH, resulting in elevated 11-oxygenated androgens (35). Furthermore, by serine phosphorylation of CYP17A1 in PCOS/PMOS (36), there is a shunting of steroid precursors towards androgens, which, together with heightened zona reticularis sensitivity to ACTH action related to elevated insulin and IGF-1 (37), amplifies androgen excess from the adrenal cortex. Additional mechanisms, including reduced cortisol-mediated suppression of ACTH secretion (38, 39) and increased peripheral conversion of androgen precursors to 11-ketotestosterone (40), in part mediated by insulin actions, worsen adrenal androgen secretion and its systemic effects in PCOS/PMOS and lead to elevated DHEA-S.

Adipose tissue is a significant site of insulin resistance in PCOS/PMOS, such that subjects ranging from lean to overweight/obese demonstrate a wide array of impairments. Abdominal adipose tissue appears to be expanded, and subcutaneous adipocytes diminished in size with hypersecretion of pro-inflammatory cytokines and attenuated secretion of adiponectin (an insulin-sensitizing cytokine) (41, 42).

Furthermore, GLUT-4 expression in adipocytes is reduced, likely in conjunction with increased miRNA-93 (43), a repressor of GLUT-4 transcription with important contributions from hyperandrogenemia (44), which impairs the phosphorylation of atypical protein kinase C in response to insulin (45). Lipid metabolism then connects these organ-specific defects into a systemic loop. Women with PCOS show metabolic inflexibility, with impaired stimulation of glucose oxidation and inadequate suppression of lipid oxidation during insulin exposure (46). Lipidomic studies demonstrate disrupted patterns of free fatty acids and phosphatidylcholines, including altered PC/lysophosphatidylcholine ratios (47). Persistent free fatty acid spillover promotes oxidative stress in liver and skeletal muscle, worsening insulin resistance and reinforcing the need for compensatory insulin secretion (48). Together, β-cell compensation, reduced insulin clearance, target-tissue resistance, and lipid spillover propel a self-reinforcing cycle that sustains hyperinsulinemia and feeds metabolic stress back onto liver and muscle (**Figure 1B**).

## 4 Hyperinsulinemia may be independent and proximate to insulin resistance

Evidence suggests hyperinsulinemia can occur independently of insulin resistance and may have a bidirectional relationship with it. In rodent studies, a high-fat diet (49) and insertion of an extra copy of the insulin gene (50) appears to induce hyperinsulinemia before onset of insulin resistance. In human studies, acute insulin infusion (51) or increased endogenous insulin secretion by insulinomas (52) are both followed by a reduction in insulin sensitivity. Sustained exposure to hyperinsulinemia has also been shown to reduce glucose uptake in skeletal muscles (53) and reduce downstream signaling (54). Regardless of the etiology of hyperinsulinemia, clinical approaches aimed at reducing insulin resistance in PCOS/PMOS may also effectively attenuate circulating insulin levels when independent pharmacotherapy to reduce hyperinsulinemia is unavailable.

## 5 Impacts of hyperinsulinemia and leptin on ovaries and the hypothalamic-pituitary axis

Ovarian cells remain selectively responsive to insulin-linked steroidogenic signals: theca cells secrete excess androgens, while granulosa-cell metabolic impairment limits follicular maturation and estradiol generation (11, 27, 55). Arrested small antral follicles then accumulate and secrete increased AMH, reducing FSH sensitivity, suppressing aromatase, and impairing dominant follicle selection (56). Collectively, elevated androgen exposure partly driven by hyperinsulinemia exposure to theca cells, attenuated FSH secretion, and elevated LH secretion contribute to ovulatory dysfunction and infertility, linking metabolic and reproductive perturbations in PCOS/PMOS.

Leptin modifies this insulin-driven ovarian dysfunction, particularly with obesity. Rather than acting primarily by changing baseline gonadotropin binding, leptin appears to interfere with insulin/IGF-I actions in ovarian steroidogenesis. In cultured human theca cells, leptin abolishes IGF-I augmentation of LH-stimulated androstenedione production. In granulosa cells, it negates IGF-I augmentation of FSH-stimulated estradiol synthesis and inhibits insulin-driven progesterone secretion (57, 58). Thus, leptin as an obesity-associated signal may worsen follicular maturation by disrupting insulin-linked ovarian endocrine-paracrine pathways in women with excess adiposity.

Metabolic hormones also act upstream of the ovary at the CNS level. Insulin stimulates GnRH gene expression in mouse GnRH neurons (59) and, in conjunction with IGF-1, augments LH secretion by the anterior pituitary (60). Whether systemic insulin resistance manifests in the human brain remains unclear. Importantly, in human studies in which insulin was acutely infused, LH secretion did not change (61). A study of PCOS hypothalamic-pituitary function (62), noted obesity-related pituitary inhibition with reduced LH secretion (63). In follow-up studies of adults with obesity, high physiological leptin concentrations were associated with reduced LH pulse amplitude, partly through increased LH clearance (64). Collectively, these data suggest that obesity modifies LH dynamics primarily at the pituitary rather than the hypothalamic level (65). In anterior pituitary cells, leptin directly stimulates LH release in a dose-dependent and potentially biphasic manner (66). Further complexity in this dynamic neuroendocrine milieu is added by hyperandrogenemia, which may directly contribute to hyperinsulinemia and systemic insulin resistance through its actions on central nervous system tissues (67). Given the complex neuroendocrine environments and signaling variability, it is unsurprising that reproductive manifestations vary within and across BMI strata, in part driven by metabolic-reproductive crosstalk.

## 6 The vicious insulin-androgen cycle and phenotype variability in PCOS

PCOS/PMOS is a heterogeneous disorder; however, the insulin-androgen positive feedback cycle is especially pronounced in patients with PCOS/PMOS and excess weight, or obesity, and biochemical evidence of insulin resistance. In most cases, insulin resistance raises insulin demand, worsening hyperinsulinemia and leading to ovarian and adrenal androgen excess. Hyperinsulinemia lowers SHBG, increasing free androgens that aggravate metabolic insulin resistance (11, 55). This loop is the clinical translation of the cellular and systemic pathways summarized in **Figure 1**.

Many women with PCOS/PMOS are overweight or obese, with prevalence reported as high as 73% in adolescents and young adults (68). Hyperandrogenemia may promote visceral adiposity and adipocyte hypertrophy, while enlarged visceral fat depots release inflammatory cytokines and leptin that further impair systemic insulin action (41, 42, 64, 69, 70). This underlies severe hepatic insulin resistance, dyslipidemia, metabolic inflexibility, and endocrine amplification in PCOS women with excess adiposity compared to their lean counterparts.

Nevertheless, lean women with PCOS/PMOS can show hyperinsulinemia, reduced insulin clearance (8), or selective tissue-level insulin signaling defects (22, 25, 71). Metabolomic studies suggest that lean women with PCOS/PMOS may differ from obesity-associated PCOS/PMOS: whereas obesity is often associated with increased lipolysis, elevated free fatty acids, and glycerol, lean PCOS may show lower glycerol and long-chain fatty acids with increased lactate, suggesting relatively preserved adipose and muscle insulin action in this group (72). These findings suggest hyperinsulinemia may stem from impaired hepatic insulin clearance or other hypersecretory mechanisms rather than systemic resistance (8, 9). There also appear to be Rotterdam phenotype (45) differences in the burden of systemic insulin resistance such that the ‘classic’ phenotypes A and B, and to a lesser extent phenotype C (ovulatory phenotype), have a disproportionate burden of insulin resistance (46, 73), while phenotype D (non-hyperandrogenic phenotype) appears to carry the lowest burden of insulin resistance (73, 74). This has been confirmed in a recent retrospective study (75).

Adiposity-related variability intersects with, but is not fully explained by, Rotterdam phenotypes used in clinical care and research (46, 76). Targeting the variable degrees of insulin resistance and hyperinsulinemia appears to be a pragmatic strategy within the evolving landscape of our understanding of PCOS/PMOS pathophysiology, and the metabolic heterogeneity central to insulin signaling in PCOS/PMOS.

## 7 Management of insulin resistance in PCOS

Lifestyle intervention combining dietary modification, structured exercise and behavioral strategies constitute the first-line treatment for PCOS/PMOS regardless of body habitus and phenotype (3, 77, 78).

Dietary interventions improve insulin sensitivity and body composition in PCOS/PMOS and have been shown to significantly improve the Homeostatic Model Assessment for Insulin Resistance (HOMA-IR), fasting insulin, fasting glucose, BMI, weight, and waist circumference, with longer intervention durations producing greater improvements (79).

Exercise augments insulin sensitivity in PCOS/PMOS through both weight-dependent and -independent mechanisms, through increases in muscle capillary density, oxidative capacity, lipid metabolism, and insulin signaling protein expression, with those having higher baseline insulin resistance benefiting the most (80, 81). Mechanistically, acute exercise leads to a fivefold increase in skeletal muscle glucose uptake through insulin-independent AMP kinase (AMPK) activation and GLUT4 translocation post-exercise. Furthermore, insulin-dependent glucose uptake remains enhanced for up to 48 hours through AMPK-mediated phosphorylation of TBC1D4 (AS160) and enhanced microvascular perfusion via nitric oxide-dependent pathways (80, 82–84). Current recommendations for exercise in PCOS/PMOS mirror those for the general population, including a minimum of 150 min/week of moderate-intensity or 75 min/week of vigorous-intensity physical activity with muscle-strengthening activities on two non-consecutive days per week, where weight loss is not indicated (3). For weight loss, ≥250 min/week of moderate-intensity or ≥150 min/week of vigorous-intensity activity, with minimization of sedentary time, is recommended (3).

Metformin remains the first-line pharmacotherapy across all PCOS/PMOS phenotypes, with higher efficacy for weight management and metabolic improvement in women with excess adiposity (3, 85). Metformin primarily suppresses hepatic glucose production by inhibiting mitochondrial complex I, reducing ATP synthesis, and increasing the AMP: ATP ratio, which activates AMPK (86, 87). AMPK shifts hepatic metabolism from anabolic pathways (e.g., lipogenesis, gluconeogenesis) toward catabolic pathways (e.g., fatty acid oxidation), thereby improving insulin sensitivity (86, 87). Metformin also blunts glucagon signaling through inhibition of adenylate cyclase and modulates gut-mediated pathways, including GLP-1 secretion and microbiota composition, thereby further enhancing glucose homeostasis (86), translating into consistent clinical benefits observed in several studies (85, 88–91). In a systematic review, metformin improved HOMA-IR, fasting glucose, and BMI versus placebo (85), a finding corroborated by a randomized trial showing this effect, in which women with obesity derived the greatest benefit (88). These effects appear to be modulated by hyperinsulinemia such that the most pronounced improvements in ovulatory cycles and hyperandrogenism occurred in hyperinsulinemic women with excess adiposity and lean women compared to their normoinsulinemic counterparts (92, 93). Supporting this notion, a hyperinsulinemic euglycemic clamp study, noted improved insulin sensitivity independent of weight change (90), with the effects persisting after treatment cessation in adolescents (91).

Glucagon-like peptide-1 receptor agonists (GLP-1RAs) improve insulin sensitivity in PCOS/PMOS through glucose-dependent insulin secretion via beta-cell cAMP signaling, reduced hepatic glucose output through glucagon suppression, and weight loss due to central appetite suppression (94). Meta-analyses demonstrate GLP-1RAs are superior to metformin for BMI reduction and HOMA-IR improvement, with combination therapy yielding additional metabolic benefits (95–97). However, current guidance recommends GLP-1RAs only as adjuncts to lifestyle intervention, due to paucity of PCOS-specific evidence, a lack of long-term safety data, and insufficient pregnancy safety information (3).

Inositol acts as a secondary messenger in insulin signaling via PI3K/Akt-mediated GLUT4 translocation. Accordingly, meta-analyses have demonstrated significant improvements in HOMA-IR, though evidence quality remains low, and current PCOS/PMOS guidelines recommend metformin over inositol (3, 98).

Thiazolidinediones, are PPARγ agonists that enhance peripheral glucose disposal, improve insulin sensitivity comparably to metformin but may cause significant weight gain (99, 100). One randomized study reported improvements in insulin action and glucose homeostasis, as well as reduced ovarian hyperandrogenism (101). Given their affordability and widespread availability, they could be considered on a case-by-case basis, especially in those with co-existing MAFLD (102).

Sodium-Glucose Cotransporter 2 (SGLT2) inhibitors promote insulin-independent urinary glucose excretion, ameliorate hyperinsulinemia and androgens, and are superior to metformin for weight reduction. However, increased genitourinary adverse events and insufficient evidence preclude routine use (103, 104), they remain promising agents.

## 8 Conclusion

PCOS/PMOS should be viewed from the prism of selective insulin resistance to integrate the reproductive and/or non-reproductive manifestations of this endocrine disorder. Hyperinsulinemia actively contributes to the phenotype by lowering SHBG, increasing free androgen bioavailability, and amplifying ovarian androgen production beyond a compensatory response to hyperglycemia (11, 28), thereby underlying variable metabolic and ovulatory dysfunction.

Obesity amplifies insulin resistance in PCOS/PMOS, but hyperinsulinemia due to impaired insulin clearance or tissue-selective signaling defects may still drive manifestations in lean women (9, 72); thus, normal fasting glucose may not exclude the role of insulin signaling in PCOS/PMOS (105).

In the clinic, metabolic risks should be evaluated across PCOS phenotypes, including oral glucose tolerance testing, lipid profiles, blood pressure, central adiposity, symptoms of obstructive sleep apnea, and screening for metabolic dysfunction-associated fatty liver disease (**Table 1**) (3). From a management perspective, structured exercise is foundational because skeletal muscle is a major site of impaired glucose disposal and contraction-mediated glucose uptake may help bypass proximal insulin signaling defects (5, 82). Metformin remains the best-established pharmacotherapy for improving insulin sensitivity and reducing hepatic glucose output, especially in patients with dysglycemia, excess adiposity, or metabolic risk factors (3, 115). Moreover, GLP-1 receptor agonists are gaining prominence for their role in weight-associated metabolic dysfunction (116), whereas inositols, thiazolidinediones, and SGLT2 inhibitors may prove useful on a case-by-case basis but require careful risk-benefit assessment (3, 117). Future studies should assess whether novel insulin-sensitizing agents, including incretin analogs, improve metabolic and reproductive dysfunction in PCOS/PMOS.

**Table 1:** Lifestyle modifications include healthy diet (no evidence to support any one type of diet composition over another), 250 min/week of moderate-intensity activities or 150 min/week of vigorous intensities for weight loss; or a minimum of 150 min/week of moderate-intensity or 75 min/week of vigorous-intensity physical activity or an equivalent combination of both for weight maintainence; plus muscle strengthening activities (eg, resistance/exibility) ideally on 2 non-consecutive days per week and behavioral strategies.

Metabolic comorbidity	Prevalence in PCOS	Screening recommendations	Management
Insulin Resistance/Prediabetes/Type 2 Diabetes	IR: 58–75%; IGT: 30–35%; T2DM: 8–10% (74, 106, 107)	75-g OGTT recommended at diagnosis (preferred over FPG/HbA1c) every 1–3 years thereafter. OGTT before pregnancy/fertility treatment (106)	Lifestyle modification* Pharmacotherapy: metformin + COCP if BMI >30 or diabetes risk factors; GLP-1RAs as adjunct for weight management (3, 106)
Obesity/Central Adiposity	50–80% obese (107)	BMI and waist circumference should be performed at each visit. Consider PCOS as additional risk factor for lower BMI threshold for bariatric surgery (3)	Lifestyle modification* Pharmacotherapy: metformin; GLP-1RAs; bariatric surgery per general population guidelines; lifestyle interventions similar effectiveness to metformin (3)
Dyslipidemia	70% (106)	Fasting lipid panel recommended at diagnosis regardless of age and BMI (3, 108)	Lifestyle modification* Pharmacotherapy: statins per ASCVD risk guidelines; manage per general population lipid guidelines (3)
Metabolic Syndrome*	33–46% in US (106, 108)	Screen all MetS components at diagnosis: waist circumference, fasting glucose/OGTT, fasting lipid panel at diagnosis regardless of age and BMI, BP; reassess annually or per risk factors (3, 106)	Lifestyle modification* Pharmacotherapy: metformin for insulin sensitization effect; statins for dyslipidemia per ASCVD risk; antihypertensives as indicated; GLP-1RAs for weight and metabolic improvement (3)
Metabolic dysfunction associated fatty liver disease (MAFLD)	35-70% (109, 110)	PCOS recognized as associated endocrine disorder for MAFLD; no specific screening recommendation in PCOS (111)	Lifestyle modification* Pharmacotherapy: metformin; GLP-1RAs (112)
Obstructive Sleep Apnea	20% (113)	Recommended screening by assessing symptoms (snoring, daytime sleepiness, waking unrefreshed); Berlin questionnaire; polysomnography if positive	Lifestyle modification* Additional therapy: CPAP therapy, GLP-1RAs per usual general population guidelines (3)

*As defined by the National Cholesterol Education Program (NCEP) Adult Treatment Panel III (ATP III) (114).

BMI, body mass index; BP, blood pressure; COCP, combined oral contraceptive pill; FPG, fasting plasma glucose; GLP-1RAs, glucagon-like peptide-1 receptor agonists; HbA1c, glycated hemoglobin; IGT, impaired glucose tolerance; IR, insulin resistance; MAFLD, metabolic dysfunction-associated fatty liver disease; MetS, metabolic syndrome; NAFLD, nonalcoholic fatty liver disease; OGTT, oral glucose tolerance test; OSA, obstructive sleep apnea; PCOS, polycystic ovary syndrome; T2DM, type 2 diabetes mellitus.
